# Dexmedetomidine for postoperative delirium in surgical patients: a mini-review of mechanisms, clinical evidence, and practical implementation

**DOI:** 10.3389/fphar.2025.1702862

**Published:** 2025-10-10

**Authors:** Xiao-yan Li, Jing-yi Yang, Yi Qiu, Cai-xia Wang, Xiao-dong Wang

**Affiliations:** Department of Anaesthesiology, The Second Affiliated Hospital of Inner Mongolia Medical University, Hohhot, China

**Keywords:** postoperative delirium, dexmedetomidine, clinical application, management, perspective

## Abstract

Postoperative delirium (POD) is a common acute neurocognitive disorder in the perioperative period, significantly increasing the risks of mortality and long-term functional decline. Dexmedetomidine, a highly selective α2-adrenergic receptor agonist, demonstrates potential for POD prevention through multimodal mechanisms, including sympatholytic effects, neuroinflammation attenuation, and physiological sleep preservation. Clinical evidence indicates its efficacy in reducing delirium incidence in cardiac surgery (risk ratio (RR), 0.57) and elderly non-cardiac surgical patients (RR, 0.51), though with notable population heterogeneity and risks of adverse effects such as bradycardia. Current guidelines recommend a dosing range of 0.1–0.7 μg/kg/h, yet monitoring requirements (e.g., electroencephalogram vs. hemodynamics) vary regionally. Future research should focus on precision dosing (e.g., biomarker-guided approaches), next-generation α2-agonists, and optimized multimodal strategies. The clinical application of dexmedetomidine requires careful risk-benefit assessment and integration into individualized perioperative protocols.

## 1 Introduction

### 1.1 Clinical significance of postoperative delirium

Postoperative delirium (POD) is an acute neurocognitive disorder marked by inattention, disorientation, and perceptual disturbances (Rudolph and Marcantonio, 2011). Its incidence ranges from 5% to 15% in general surgery to 50% in high-risk groups like elderly and cardiac surgery patients (Li et al., 2023). Major risk factors include preexisting cognitive impairment, polypharmacy, and comorbidities (Shi et al., 2019).

POD significantly worsens clinical outcomes, prolonging hospitalization by 3–5 days (Park and Kim, 2019) and increasing 30-day mortality (odds ratio (OR), 1.9) (Shi et al., 2019). It also predicts long-term functional decline, with persistent impairments in activities of daily living (Aldwikat et al., 2023). Furthermore, 30% of patients develop lasting cognitive dysfunction, accelerating dementia progression (Shi et al., 2019). These findings highlight POD as a critical perioperative challenge requiring targeted prevention strategies.

### 1.2 Limitations of current treatment strategies

Current approaches to POD management present significant limitations in both pharmacological and non-pharmacological domains. Pharmacological interventions remain controversial, with emerging evidence suggesting that commonly used sedatives may paradoxically increase delirium risk. A recent prospective cohort study in intensive care unit (ICU) patients reported that sedation with midazolam, compared with propofol, was associated with a higher risk of delirium, underscoring the potential role of midazolam in increasing POD incidence in vulnerable population (van Gelder et al., 2024). Similarly, systematic reviews suggest that while benzodiazepines provide effective sedation, their GABAergic mechanism may exacerbate neurocognitive dysfunction (Wang et al., 2023; Makaron et al., 2013). Notably, a meta-analysis by Wang et al. found that benzodiazepines were associated with a higher risk of POD specifically when compared directly to dexmedetomidine (Wang et al., 2023), underscoring a context-dependent risk profile. Other reviews also highlight potential neurocognitive risks (Makaron et al., 2013). Opioid analgesics, though essential for pain management, show dose-dependent associations with delirium development in vulnerable populations (Yoo et al., 2024).

Non-pharmacological strategies, particularly the ABCDEF bundle, face substantial implementation challenges despite strong theoretical foundations. Observational studies reveal adherence rates below 60% in critical care settings, primarily due to staffing limitations and workflow disruptions (DeMellow et al., 2020). A comprehensive meta-analysis confirmed these interventions’ variable effectiveness, noting particular shortcomings in emergency surgical contexts and resource-constrained environments (Sosnowski et al., 2023). This creates a clinical paradox where pharmacological options may inadvertently worsen delirium while behavioral interventions prove difficult to consistently implement.

### 1.3 Potential value of dexmedetomidine

Dexmedetomidine demonstrates unique pharmacological advantages for POD prevention through its multimodal mechanism of action. As a selective α2-adrenoreceptor agonist, it modulates sympathetic activity by inhibiting norepinephrine release from the locus coeruleus, thereby attenuating the stress response implicated in delirium pathogenesis (Zeng et al., 2019). Its anti-inflammatory properties are particularly relevant, with meta-analyses confirming significant reductions in pro-inflammatory cytokines including interleukin (IL)-6 and tumor necrosis factor-alpha TNF-α when used as an anesthetic adjunct (Li et al., 2015).

Clinically, dexmedetomidine shows superior neurocognitive outcomes compared to traditional sedatives. A randomized controlled trial by Skrobik et al. demonstrated its efficacy in preventing ICU delirium when administered at low nocturnal doses (0.1–0.2 μg/kg/h) (Skrobik et al., 2018). Preclinical studies further support its neuroprotective effects, with Sun et al. identifying specific protection against hippocampal neuron apoptosis through epigenetic regulation of the miR-129/YAP1/JAG1 axis (Sun et al., 2020).

The drug’s ability to preserve natural sleep architecture while avoiding anticholinergic and GABAergic side effects (common with benzodiazepines) represents a significant therapeutic advance (Zeng et al., 2019). These properties collectively position dexmedetomidine as a promising alternative for high-risk surgical populations, though optimal dosing strategies and long-term cognitive benefits require further investigation.

## 2 Mechanistic insights

### 2.1 Pathophysiological basis of delirium

**FIGURE 1:**
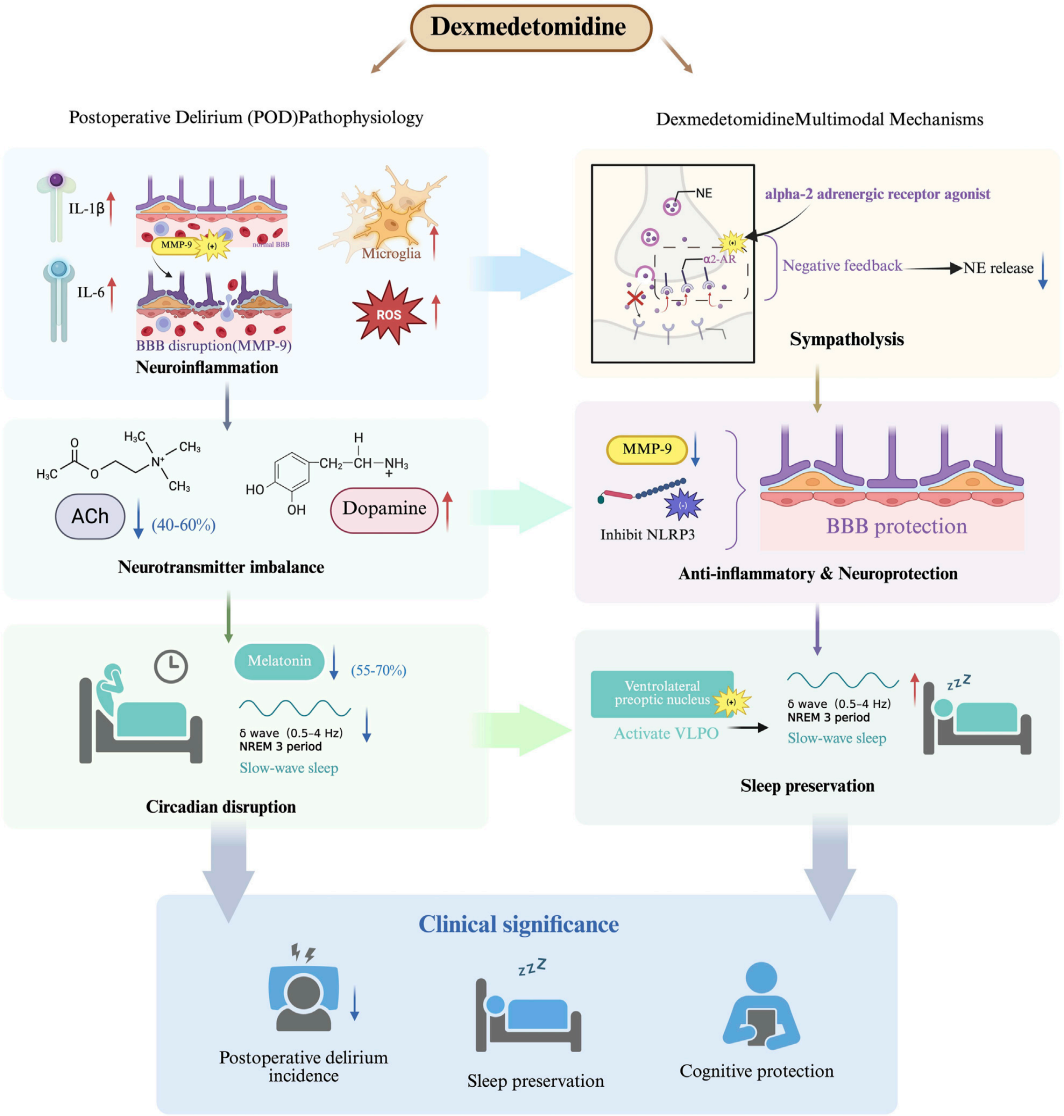
Mechanisms of dexmedetomidine in preventing postoperative delirium.

POD arises from three interrelated pathological processes that collectively disrupt neural network function (Figure 1). First, the neuroinflammatory cascade triggered by surgical trauma leads to peripheral cytokine release (IL-1β, IL-6) and subsequent blood-brain barrier dysfunction through matrix metalloproteinase-9 (MMP-9) mediated tight junction degradation (Liu et al., 2022) This inflammatory response activates microglia, generating reactive oxygen species that impair neuronal mitochondrial function (Subramaniyan and Terrando, 2019). Second, neurotransmitter system dysregulation manifests as a characteristic cholinergic deficiency, with cerebrospinal fluid acetylcholine levels decreasing by 40%–60% postoperatively (Hshieh et al., 2008). This occurs concomitantly with dopaminergic hyperactivity in mesolimbic pathways, creating an imbalance that disrupts cortical integration and attentional processing. Third, circadian rhythm disruption emerges through suprachiasmatic nucleus dysfunction, characterized by significant melatonin suppression (55%–70% reduction) and impaired slow-wave sleep generation (Campbell and Figueiro, 2024). These mechanisms form a self-reinforcing cycle of neural instability that clinically manifests as the characteristic symptoms of delirium.

### 2.2 Multimodal mechanisms of dexmedetomidine in delirium prevention

Dexmedetomidine exhibits a unique multimodal pharmacological profile that addresses several pathophysiological pathways implicated in POD (Figure 1). The drug’s high selectivity for α2-adrenergic receptors (α2:α1 binding ratio >1600:1) enables targeted modulation of sympathetic outflow from the locus coeruleus, with clinical studies demonstrating significant reductions in norepinephrine release during surgical procedures (Kang et al., 2020; Gertler et al., 2001). This sympatholytic effect is particularly relevant given the established association between sympathetic hyperactivity and delirium pathogenesis, while the preservation of partial adrenergic tone maintains cardiovascular stability better than complete sympathetic blockade.

The neuroprotective properties of dexmedetomidine extend beyond sympathetic modulation to include significant anti-inflammatory effects (Figure 1). Experimental evidence indicates the drug attenuates neuroinflammation through multiple mechanisms, including inhibition of NOD-like receptor family, pyrin domain-containing 3 inflammasome assembly (62.4% reduction) and downregulation of MMP-9 activity in hippocampal microvasculature (Hu et al., 2022). These actions help preserve blood-brain barrier integrity and reduce neuronal injury from inflammatory mediators that are strongly correlated with delirium severity.

Unlike conventional sedatives that disrupt normal sleep architecture, dexmedetomidine demonstrates the unique ability to enhance slow-wave sleep duration through selective modulation of ventrolateral preoptic nucleus neurons (Oto et al., 2012). This preservation of physiological sleep patterns is clinically significant given the independent association between slow-wave sleep deficiency and increased delirium risk (OR, 3.2). Comparative clinical trials have demonstrated dexmedetomidine’s superiority over benzodiazepines in reducing acute brain dysfunction, with the MENDS randomized controlled trial showing significant reductions in delirium prevalence and duration (Pandharipande et al., 2007). The drug’s favorable pharmacological profile-including minimal anticholinergic effects and preserved respiratory drive - positions it as an optimal choice for high-risk surgical populations where delirium prevention is paramount.

## 3 Clinical evidence appraisal: dexmedetomidine for POD prevention

### 3.1 Supportive clinical trial evidence in POD prevention

Current evidence demonstrates dexmedetomidine’s variable efficacy in POD prevention across different surgical populations (Table 1). In cardiac surgery, findings remain inconclusive - while the large DECADE trial (n = 794) showed no significant delirium reduction with dexmedetomidine (17% vs. 12% placebo) (Turan et al., 2020), a subsequent network meta-analysis of 18 trials (n = 2,636) revealed postoperative administration significantly lowered delirium risk versus placebo (OR, 0.13; 95% confidence interval (CI), 0.03–0.35) and propofol (OR, 0.19; 95% CI, 0.04–0.66) (Shang et al., 2023). This discrepancy highlights the importance of timing in dexmedetomidine administration for cardiac surgical patients.

**TABLE 1:** Summary of clinical evidence and applications of dexmedetomidine for POD prevention.

Patient population	Study type	Intervention	Main findings	References
Cardiac surgery patients (n = 794)	RCT (DECADE Trial)	Intraoperative dexmedetomidine vs. placebo	No significant reduction in POD (17% vs. 12%)	Turan et al. (2020)
Cardiac surgery patients (n = 2,636)	Network meta-analysis (18 RCTs)	Postoperative dexmedetomidine vs. placebo/propofol	Significantly reduced POD risk (OR 0.13 vs. placebo; OR 0.19 vs. propofol)	Shang et al. (2023)
Elderly non-cardiac surgery patients (n = 2,102)	Meta-analysis (6 RCTs)	Intraoperative dexmedetomidine	Reduced POD risk (RR 0.61) and fewer cardiovascular complications	Zeng et al. (2019)
ICU patients requiring prolonged ventilation	RCT (MIDEX/PRODEX)	Dexmedetomidine vs. midazolam/propofol	Reduced ventilation duration (↓41 h), improved patient interaction, but increased bradycardia	Jakob et al. (2012)
Elderly non-cardiac surgery patients (n = 700)	Multicenter RCT	Dexmedetomidine 0.1 μg/kg/h	POD incidence significantly reduced (23% → 9%), especially in cognitively vulnerable patients	Su et al. (2016)
Cardiac surgery patients	Meta-analysis	Perioperative dexmedetomidine	Reduced POD risk by 25% (OR 0.75); significantly decreased IL-6 levels	Meng et al. (2025)
Elderly non-cardiac surgery patients	RCT	Intraoperative dexmedetomidine	POD risk halved (RR 0.53); early intervention particularly effective	Li et al. (2020a)
Cardiac surgery patients	Systematic review and meta-analysis (16 RCTs)	Perioperative dexmedetomidine	POD risk reduced by 43% (RR 0.57)	Zhuang et al. (2024)
Elderly patients under regional anesthesia	Meta-analysis (18 RCTs)	Intravenous dexmedetomidine	POD risk reduced by 52% (RR 0.48)	Wang et al. (2024a)
Elderly non-cardiac surgery patients	Systematic review and meta-analysis (16 studies)	Perioperative dexmedetomidine	POD risk reduced by 49% (RR 0.51)	Shen et al. (2020)

POD, postoperative delirium; RCT, randomized controlled trial; ICU, intensive care unit; RR, risk ratio; OR, odds ratio; IL-6, Interleukin-6.

For elderly patients undergoing non-cardiac surgery, stronger evidence supports dexmedetomidine’s benefits. A meta-analysis of 6 randomized controlled trials (RCTs) (n = 2,102) demonstrated significant delirium reduction (risk ratio (RR), 0.61; 95% CI, 0.34–0.76) alongside decreased cardiovascular complications (Zeng et al., 2019). The drug’s favorable safety profile in this vulnerable population makes it particularly valuable for non-cardiac procedures where delirium risk remains high but cardiac complications are less prevalent.

In critical care settings, dexmedetomidine shows distinct advantages over traditional sedatives. The MIDEX-PRODEX trials established its superiority in prolonged mechanical ventilation, reducing median ventilation duration by 41 h versus midazolam (123 vs. 164 h, *P* = 0.03) while improving patient interaction scores (Jakob et al., 2012). These benefits likely stem from dexmedetomidine’s unique ability to maintain physiological sleep patterns and reduce electroencephalographic disruption compared to GABAergic alternatives, though its higher rates of bradycardia and hypotension require careful patient selection and monitoring.

### 3.2 Controversial findings in dexmedetomidine research

The efficacy of dexmedetomidine for POD prevention remains contested despite promising mechanistic data (Table 1). The multicenter trial by Deiner et al. represents the largest randomized controlled study to date, demonstrating equivalent delirium rates between dexmedetomidine (12.2%) and placebo (11.4%) groups in elderly non-cardiac surgery patients (*P* = 0.94) (Deiner et al., 2017). This null finding contrasts sharply with positive results from ICU-based studies, suggesting the drug’s effects may be context-dependent, particularly regarding administration timing and surgical population characteristics.

Pharmacokinetic studies provide critical insights into these disparate outcomes. Weerink et al. identified substantial inter-individual variability in drug metabolism, with subtherapeutic plasma concentrations (<0.2 ng/mL) occurring in nearly 40% of patients at standard dosing (Weerink et al., 2017). Furthermore, Ebert et al. characterized dexmedetomidine’s narrow therapeutic window (0.2–0.6 ng/mL) (Ebert et al., 2000), beyond which dose-dependent hypotension and bradycardia may outweigh potential neurological benefits. These pharmacological properties may explain the trial’s neutral results while suggesting opportunities for optimized dosing strategies.

Clinical implementation challenges further complicate dexmedetomidine’s role in delirium prevention. As noted by Vacas and Van de Wiele (2017), the drug’s hemodynamic effects require careful patient selection and monitoring, particularly in elderly populations with cardiovascular comorbidities. These considerations underscore the need for precision medicine approaches that account for individual pharmacokinetic variability, comorbid medication use, and surgical context when considering dexmedetomidine for neuroprotection.

### 3.3 Population-specific treatment effects

The efficacy of dexmedetomidine for POD prevention exhibits significant variation across patient populations, necessitating careful consideration of clinical context (Table 1). In elderly patients undergoing non-cardiac surgery, the landmark RCT by Su et al. demonstrated particularly robust benefits, with dexmedetomidine infusion (0.1 μg/kg/h) reducing delirium incidence from 23% to 9% (OR, 0.35; 95% CI, 0.22–0.54; *P* < 0.0001) (Su et al., 2016). This protective effect was most pronounced in patients with preoperative cognitive vulnerability, suggesting particular utility in high-risk geriatric populations.

Cardiac surgery patients represent another responsive subgroup, as evidenced by multiple meta-analyses. Meng et al. reported a 25% relative risk reduction (OR, 0.75; 95% CI, 0.57–0.98) (Meng et al., 2025), while Li et al. found even greater benefits (OR, 0.56; 95% CI, 0.36–0.89) (Li et al., 2021), particularly with postoperative administration. These findings likely reflect dexmedetomidine’s ability to mitigate cardiopulmonary bypass-induced neuroinflammation and sympathetic activation, mechanisms particularly relevant in cardiac surgical settings.

However, clinical benefits appear context-dependent, with notable limitations in certain populations. The multicenter trial by Deiner et al. found no significant delirium reduction with intraoperative dexmedetomidine in major non-cardiac surgery (12.2% vs. 11.4%, *P* = 0.94) (Deiner et al., 2017), highlighting the importance of administration timing. Furthermore, subgroup analyses suggest limited efficacy in emergency surgeries and patients with advanced dementia, emphasizing the need for careful patient selection and protocol optimization in clinical practice.

### 3.4 Evidence synthesis and clinical implications

Current evidence demonstrates dexmedetomidine’s efficacy in POD prevention when administered intraoperatively at 0.1–0.5 μg/kg/h. A meta-analysis of cardiac surgery patients showed a 25% reduction in delirium incidence (OR, 0.75) with dexmedetomidine use (Meng et al., 2025). The drug’s anti-inflammatory effects are particularly notable, significantly reducing IL-6 levels by 25.14 pg/mL immediately post-operation (Li et al., 2015). In elderly non-cardiac surgical patients, intraoperative dexmedetomidine administration halved delirium risk (RR, 0.53), highlighting the importance of early intervention (Li CJ. et al., 2020). These findings support dexmedetomidine’s role in multimodal prevention strategies for high-risk surgical populations.

## 4 Practical recommendations for clinical application

### 4.1 Target patient populations

Current evidence identifies three high-risk surgical populations that derive significant benefit from dexmedetomidine administration for POD prevention (Table 1). First, cardiac surgery patients undergoing cardiopulmonary bypass demonstrate a 43% relative risk reduction (RR, 0.57; 95% CI, 0.41–0.79) when receiving perioperative dexmedetomidine, as shown in a systematic review of 16 randomized trials (Zhuang et al., 2024). Second, elderly patients (≥60 years) receiving regional anesthesia exhibit a 52% lower delirium incidence (RR, 0.48; 95% CI, 0.37–0.63) with intraoperative dexmedetomidine infusion, according to a meta-analysis of 18 RCTs (Wang et al., 2024a). Third, elderly non-cardiac surgical patients show comparable benefits, with a 49% risk reduction (RR, 0.51; 95% CI, 0.43–0.61) across 16 included trials (Shen et al., 2020).

Clinical application requires careful patient selection due to potential hemodynamic effects. Dexmedetomidine is contraindicated in patients with hemodynamically significant bradycardia, high-grade atrioventricular block, or decompensated shock requiring vasopressor support (Reel and Maani, 2025). Particular caution is warranted in patients with severe hepatic impairment (Child-Pugh C), where reduced dosing may be necessary due to altered pharmacokinetics. These evidence-based recommendations support targeted use of dexmedetomidine while emphasizing the importance of individualized risk-benefit assessment.

### 4.2 Dosing regimen optimization

Current evidence supports weight-based dexmedetomidine dosing for delirium prevention, with optimal regimens varying by surgical context and patient characteristics. For intraoperative initiation in non-cardiac surgery, a maintenance infusion of 0.1–0.5 μg/kg/h without loading dose provides effective delirium prevention while minimizing hemodynamic instability, as demonstrated in a meta-analysis of 18 randomized trials (Wang et al., 2024b). In cardiac surgery populations, lower infusion rates (0.1–0.5 μg/kg/h) show superior cognitive outcomes compared to higher doses (0.5–0.9 μg/kg/h), with significantly reduced incidence of hypotension (7.3% vs. 31.6%, *P* = 0.044) (Fang et al., 2023). These findings suggest a therapeutic window exists where adequate α2-adrenoreceptor activation is achieved without excessive sympathetic inhibition.

Postoperative ICU management requires careful dose titration, particularly in elderly patients. A multicenter trial of cardiac surgery patients found overnight dexmedetomidine infusion (8p.m.-8a.m.) failed to reduce delirium incidence (12.6% vs. 12.4%, *P* = 0.97) while significantly increasing hypotensive events (7.3% vs. 0.6%, *P* < 0.01) (Huet et al., 2024). Pharmacodynamic studies in elderly patients under spinal anesthesia identified an ED95 of 0.86 μg/kg for adequate sedation, but noted doses exceeding 0.5 μg/kg substantially increase hypotension risk (31.6% vs. 3.6%, *P* = 0.013) (Ko et al., 2015). Similarly, research in hip replacement surgery demonstrated loading doses ≥0.5 μg/kg cause significant mean arterial pressure reductions at multiple perioperative time points (*P* < 0.05) (Liu et al., 2023).

Risk stratification is essential for safe administration. A retrospective analysis of 283 ICU patients identified three independent predictors of dexmedetomidine-associated hypotension: baseline mean arterial pressure (OR, 0.97; 95% CI, 0.95–0.99), Acute Physiology and Chronic Health Evaluation II scores (OR, 1.06; 95% CI, 1.01–1.12), and coronary artery disease (OR, 0.48; 95% CI, 0.26–0.90) (Gerlach et al., 2016). These findings support initiating therapy at 0.1–0.3 μg/kg/h in high-risk populations, with gradual titration guided by continuous hemodynamic monitoring. For elderly patients (≥65 years) and those with cardiovascular comorbidities, additional precautions including extended loading infusion duration (30 min) and 25% dose reduction should be considered to balance efficacy and safety.

### 4.3 Multimodal integration strategies

Effective delirium management necessitates a synergistic approach combining pharmacological and non-pharmacological interventions for optimal delirium prevention. The modified Hospital Elder Life Program demonstrates particular efficacy, reducing delirium incidence by 56% (RR, 0.44; 95% CI, 0.23–0.83) in elderly surgical patients through structured cognitive stimulation, early mobilization, and nutritional support (Chen et al., 2017). Pharmacologically, dexmedetomidine emerges as the preferred sedative, particularly when combined with low-dose ketamine (0.15–0.3 mg/kg/h), which reduces delirium incidence by 43% compared to standard regimens (Perbet et al., 2018). Importantly, benzodiazepines should be avoided in high-risk patients due to their association with prolonged delirium duration (RR, 1.64; 95% CI, 1.27–2.10) (Pisani et al., 2009).

Implementation requires standardized protocols incorporating: (1) preoperative risk assessment, (2) intraoperative dexmedetomidine for high-risk cases, (3) postoperative avoidance of deliriogenic medications, and (4) immediate cognitive/physical rehabilitation. As demonstrated in a stepped-wedge trial, such multimodal programs significantly reduce both delirium incidence (OR, 0.87) and delirium days (5.3% vs. 6.9%) (Deeken et al., 2022). This comprehensive approach addresses multiple pathogenic pathways while minimizing adverse effects, representing the current standard for perioperative delirium prevention.

## 5 Challenges and future perspectives in dexmedetomidine application for POD

### 5.1 Critical unresolved clinical questions

Several critical knowledge gaps persist regarding the optimal use of dexmedetomidine for delirium prevention. First, the temporal relationship between administration timing and clinical outcomes remains incompletely characterized, with insufficient comparative data to determine whether preoperative, intraoperative, or postoperative initiation yields superior results. Second, the durability of neuroprotective effects beyond 3 years post-intervention requires clarification, as current evidence demonstrates conflicting results regarding long-term cognitive preservation (Hong et al., 2025; Zhang et al., 2019). Third, the medication’s impact on patients with pre-existing cognitive impairment warrants further investigation, particularly given the established association between delirium and accelerated cognitive decline in vulnerable populations (Gross et al., 2012). Fourth, precise dose-response relationships for both short-term delirium prevention and long-term neurocognitive outcomes have not been adequately defined across different surgical populations and age groups. Finally, population-specific efficacy thresholds need elucidation, including optimal dosing strategies for cardiac versus non-cardiac surgery patients and variations based on baseline risk profiles. These unresolved issues highlight the need for standardized, large-scale longitudinal studies with comprehensive cognitive assessments to establish evidence-based protocols for clinical practice.

### 5.2 Promising research directions

Several critical research priorities are emerging to advance the clinical application of dexmedetomidine for neuroprotection. First, rigorous validation of predictive biomarkers represents an urgent need, particularly for establishing reliable thresholds of neurofilament light chain and other neuronal injury markers that can guide patient stratification (Hou et al., 2023). Second, the clinical translation of novel delivery systems requires systematic evaluation, including pharmacokinetic studies of the multivalent bioadhesive nanoparticle clusters demonstrated by Jia et al. (2025) and safety assessments of alternative administration routes characterized by Jeong et al. (2023). Third, the development of dynamic dosing algorithms must incorporate multiple data streams including real-time physiological monitoring, individual pharmacokinetic profiles from therapeutic drug monitoring, and comprehensive surgical risk factor analysis. Fourth, pharmacological research should focus on next-generation α2-agonists with improved receptor subtype selectivity to enhance therapeutic effects while minimizing adverse hemodynamic consequences. Finally, the integration of artificial intelligence platforms offers transformative potential for adaptive treatment optimization, though this requires validation through prospective clinical trials comparing algorithm-guided dosing versus standard protocols. These priorities collectively address the key gaps in moving from current empirical approaches to precision medicine paradigms for postoperative neuroprotection, while maintaining rigorous standards of evidence generation and clinical safety evaluation.

### 5.3 Contemporary guideline landscape

Current guidelines demonstrate regional variations in dexmedetomidine recommendations for delirium prevention. The American Geriatrics Society (AGS) provides conditional support (Grade 2B), favoring ICU use as a GABAergic alternative while prioritizing non-pharmacologic interventions (American Geriatrics Society Expert Panel on Postoperative Delirium in Older Adults, 2015; Hebert, 2018). In contrast, Chinese consensus advocates broader perioperative application (Grade B), particularly for elderly surgical patients, reflecting population-specific risk-benefit assessments (Li M. et al., 2020). The recently updated Society of Critical Care Medicine guidelines further delineate its role, reinforcing the integration of dexmedetomidine into multimodal sedation-analgesia protocols to mitigate delirium risk in critically ill adults (Lewis et al., 2025).

Both guidelines agree on dose limits (<0.7 μg/kg/h) but differ in monitoring: AGS emphasizes electroencephalographic for neurocognitive safety, while Asian protocols focus on hemodynamic stability (American Geriatrics Society Expert Panel on Postoperative Delirium in Older Adults, 2015; Li M. et al., 2020). These differences highlight how clinical context influences guideline development, with AGS aligning with geriatric deprescribing principles and Asian consensus addressing higher baseline delirium risks. Multidisciplinary care and preoperative risk stratification remain universal recommendations.

## 6 Summary

Dexmedetomidine demonstrates clinically significant benefits for POD prevention in high-risk surgical populations, particularly elderly cardiac surgery patients. Its multimodal mechanism—combining sympatholytic, anti-inflammatory, and sleep-preserving properties—offers advantages over traditional sedatives. Current evidence supports weight-based dosing (0.1–0.3 μg/kg/h) with hemodynamic monitoring, while contraindications include conduction abnormalities and hemodynamic instability. However, this review acknowledges limitations in analytical depth regarding mechanistic pathways and clinical evidence integration, which may affect the comprehensiveness of the conclusions. Future iterations should strengthen the translational perspective by incorporating more robust biomarker-guided mechanistic analyses and broader clinical validation across diverse surgical settings. Key knowledge gaps persist regarding long-term cognitive outcomes, optimal timing of administration, and population-specific efficacy. Future research should focus on next-generation α2-agonists, biomarker-guided precision dosing, and standardized trials with neuropsychological assessments. Additionally, more comprehensive synthesis of real-world evidence and comparative effectiveness data will be essential to establish standardized, evidence-based clinical guidelines. Clinicians should employ dexmedetomidine judiciously within evidence-based protocols while contributing to outcomes research to refine its role in perioperative neuroprotection.

## Abbreviations

POD, postoperative delirium; OR, odds ratio; GABAergic, γ-Aminobutyric Acid-ergic; AGS, American Geriatrics Society; ICU, intensive care unit; IL, interleukin; MMP-9, matrix metalloproteinase-9; CI, confidence interval; RCTs, randomized controlled trials; RR, risk ratio.

## References

[B1] Aldwikat R. K. Manias E. Holmes A. C. Tomlinson E. Nicholson P. (2023). Associations of postoperative delirium with activities of daily living in older people after major surgery: a prospective cohort study. J. Clin. Nurs. 32 (19-20), 7578–7588. 10.1111/jocn.16801 37341067

[B2] American Geriatrics Society Expert Panel on Postoperative Delirium in Older Adults (2015). American geriatrics society abstracted clinical practice guideline for postoperative delirium in older adults. J. Am. Geriatr. Soc. 63 (1), 142–150. 10.1111/jgs.13281 25495432 PMC5901697

[B3] Campbell E. Figueiro M. G. (2024). Postoperative cognitive dysfunction: spotlight on light, circadian rhythms, and sleep. Front. Neurosci. 18, 1390216. 10.3389/fnins.2024.1390216 38699675 PMC11064652

[B4] Chen C. C. Li H. C. Liang J. T. Lai I. R. Purnomo J. D. T. Yang Y. T. (2017). Effect of a modified hospital Elder Life Program on delirium and length of hospital stay in patients undergoing abdominal surgery: a cluster randomized clinical trial. JAMA Surg. 152 (9), 827–834. 10.1001/jamasurg.2017.1083 28538964 PMC5710459

[B5] Deeken F. Sánchez A. Rapp M. A. Denkinger M. Brefka S. Spank J. (2022). Outcomes of a delirium prevention Program in older persons after elective surgery: a stepped-wedge cluster randomized clinical trial. JAMA Surg. 157 (2), e216370. 10.1001/jamasurg.2021.6370 34910080 PMC8674802

[B6] Deiner S. Luo X. Lin H. M. Sessler D. I. Saager L. Sieber F. E. (2017). Intraoperative infusion of dexmedetomidine for prevention of postoperative delirium and cognitive dysfunction in elderly patients undergoing major elective noncardiac surgery: a randomized clinical trial. JAMA Surg. 152 (8), e171505. 10.1001/jamasurg.2017.1505 28593326 PMC5831461

[B7] DeMellow J. M. Kim T. Y. Romano P. S. Drake C. Balas M. C. (2020). Factors associated with ABCDE bundle adherence in critically ill adults requiring mechanical ventilation: an observational design. Intensive Crit. Care Nurs. 60, 102873. 10.1016/j.iccn.2020.102873 32414557 PMC7988688

[B8] Ebert T. J. Hall J. E. Barney J. A. Uhrich T. D. Colinco M. D. (2000). The effects of increasing plasma concentrations of dexmedetomidine in humans. Anesthesiology 93 (2), 382–394. 10.1097/00000542-200008000-00016 10910487

[B9] Fang J. Yang J. Zhai M. Zhang Q. Zhang M. Xie Y. (2023). Effects of dexmedetomidine dosage on the short-term cognitive function of elderly patients undergoing cardiac surgery. BMC Anesthesiol. 23 (1), 380. 10.1186/s12871-023-02315-6 37985971 PMC10658921

[B10] Gerlach A. T. Blais D. M. Jones G. M. Burcham P. K. Stawicki S. P. Cook C. H. (2016). Predictors of dexmedetomidine-associated hypotension in critically ill patients. Int. J. Crit. Illn. Inj. Sci. 6 (3), 109–114. 10.4103/2229-5151.190656 27722111 PMC5051052

[B11] Gertler R. Brown H. C. Mitchell D. H. Silvius E. N. (2001). Dexmedetomidine: a novel sedative-analgesic agent. Proc. Bayl Univ. Med. Cent. 14 (1), 13–21. 10.1080/08998280.2001.11927725 16369581 PMC1291306

[B12] Gross A. L. Jones R. N. Habtemariam D. A. Fong T. G. Tommet D. Quach L. (2012). Delirium and long-term cognitive trajectory among persons with dementia. Arch. Intern Med. 172 (17), 1324–1331. 10.1001/archinternmed.2012.3203 23403619 PMC3740440

[B13] Hebert C. (2018). Evidence-Based practice in Perianesthesia nursing: application of the American Geriatrics Society Clinical Practice Guideline for postoperative delirium in older adults. J. Perianesth Nurs. 33 (3), 253–264. 10.1016/j.jopan.2016.02.011 29784254

[B14] Hong H. Li X. Yang J. Zhang Y. Liu G. Y. Yan F. X. (2025). Impact of perioperative dexmedetomidine on long-term outcomes in older patients following cardiac surgery: follow-up of a randomized trial. BMC Anesthesiol. 25 (1), 130. 10.1186/s12871-025-02963-w 40097932 PMC11912700

[B15] Hou Y. R. Xu C. Y. An M. Z. Li Z. P. Ni H. D. Chen T. (2023). Effect of dexmedetomidine on postoperative plasma neurofilament light chain in elderly patients undergoing thoracoscopic surgery: a prospective, randomized controlled trial. Clin. Interv. Aging 18, 1565–1576. 10.2147/CIA.S422560 37727450 PMC10506605

[B16] Hshieh T. T. Fong T. G. Marcantonio E. R. Inouye S. K. (2008). Cholinergic deficiency hypothesis in delirium: a synthesis of current evidence. J. Gerontol. A Biol. Sci. Med. Sci. 63 (7), 764–772. 10.1093/gerona/63.7.764 18693233 PMC2917793

[B17] Hu Y. Zhou H. Zhang H. Sui Y. Zhang Z. Zou Y. (2022). The neuroprotective effect of dexmedetomidine and its mechanism. Front. Pharmacol. 13, 965661. 10.3389/fphar.2022.965661 36204225 PMC9531148

[B18] Huet O. Gargadennec T. Oilleau J. F. Rozec B. Nesseler N. Bouglé A. (2024). Prevention of post-operative delirium using an overnight infusion of dexmedetomidine in patients undergoing cardiac surgery: a pragmatic, randomized, double-blind, placebo-controlled trial. Crit. Care 28 (1), 64. 10.1186/s13054-024-04842-1 38419119 PMC10902989

[B19] Jakob S. M. Ruokonen E. Grounds R. M. Sarapohja T. Garratt C. Pocock S. J. (2012). Dexmedetomidine vs midazolam or propofol for sedation during prolonged mechanical ventilation: two randomized controlled trials. JAMA 307 (11), 1151–1160. 10.1001/jama.2012.304 22436955

[B20] Jeong S. H. Jang J. H. Lee Y. B. (2023). Drug delivery to the brain *via* the nasal route of administration: exploration of key targets and major consideration factors. J. Pharm. Investig. 53 (1), 119–152. 10.1007/s40005-022-00589-5 35910081 PMC9308891

[B21] Jia Y. Kong X. Li R. Wang H. Li C. Cheng S. (2025). Enhanced nasal-to-brain drug delivery by multivalent bioadhesive nanoparticle clusters for cerebral ischemic reperfusion injury protection. Acta Biomater. 194, 411–427. 10.1016/j.actbio.2025.01.036 39870153

[B22] Kang R. Jeong J. S. Ko J. S. Lee S. Y. Lee J. H. Choi S. J. (2020). Intraoperative dexmedetomidine attenuates norepinephrine levels in patients undergoing transsphenoidal surgery: a randomized, placebo-controlled trial. BMC Anesthesiol. 20 (1), 100. 10.1186/s12871-020-01025-7 32359367 PMC7195722

[B23] Ko K. H. Jun I. J. Lee S. Lim Y. Yoo B. Kim K. M. (2015). Effective dose of dexmedetomidine to induce adequate sedation in elderly patients under spinal anesthesia. Korean J. Anesthesiol. 68 (6), 575–580. 10.4097/kjae.2015.68.6.575 26634081 PMC4667143

[B24] Lewis K. Balas M. C. Stollings J. L. McNett M. Girard T. D. Chanques G. (2025). A focused update to the Clinical Practice Guidelines for the prevention and management of pain, anxiety, Agitation/Sedation, delirium, immobility, and sleep disruption in adult patients in the ICU. Crit. Care Med. 53 (3), e711–e727. 10.1097/CCM.0000000000006574 39982143

[B25] Li B. Li Y. Tian S. Wang H. Wu H. Zhang A. (2015). Anti-inflammatory effects of perioperative dexmedetomidine administered as an adjunct to general Anesthesia: a meta-analysis. Sci. Rep. 5, 12342. 10.1038/srep12342 26196332 PMC4508837

[B26] Li C. J. Wang B. J. Mu D. L. Hu J. Guo C. Li X. Y. (2020a). Randomized clinical trial of intraoperative dexmedetomidine to prevent delirium in the elderly undergoing major non-cardiac surgery. Br. J. Surg. 107 (2), e123–e132. 10.1002/bjs.11354 31903588

[B27] Li M. Wang T. L. Wang D. X. (2020b). An overview of Chinese multidisciplinary expert consensus on perioperative brain health in elderly patients. Chin. Med. J. Engl. 134 (1), 5–7. 10.1097/CM9.0000000000001213 33109953 PMC7862808

[B28] Li P. Li L. X. Zhao Z. Z. Xie J. Zhu C. L. Deng X. M. (2021). Dexmedetomidine reduces the incidence of postoperative delirium after cardiac surgery: a meta-analysis of randomized controlled trials. BMC Anesthesiol. 21 (1), 153. 10.1186/s12871-021-01370-1 34006239 PMC8130348

[B29] Li S. Li R. Li M. Cui Q. Zhang X. Ma T. (2023). Dexmedetomidine administration during brain tumour resection for prevention of postoperative delirium: a randomised trial. Br. J. Anaesth. 130, e307–e316. 10.1016/j.bja.2022.10.041 36517290

[B30] Liu L. F. Hu Y. Liu Y. N. Shi D. W. Liu C. Da X. (2022). Reactive oxygen species contribute to delirium-like behavior by activating CypA/MMP9 signaling and inducing blood-brain barrier impairment in aged mice following anesthesia and surgery. Front. Aging Neurosci. 14, 1021129. 10.3389/fnagi.2022.1021129 36337710 PMC9629746

[B31] Liu H. Gao M. Zheng Y. Sun C. Lu Q. Shao D. (2023). Effects of dexmedetomidine at different dosages on perioperative haemodynamics and postoperative recovery quality in elderly patients undergoing hip replacement surgery under general anaesthesia: a randomized controlled trial. Trials 24 (1), 386. 10.1186/s13063-023-07384-z 37291651 PMC10249318

[B32] Makaron L. Moran C. A. Namjoshi O. Rallapalli S. Cook J. M. Rowlett J. K. (2013). Cognition-impairing effects of benzodiazepine-type drugs: role of GABAA receptor subtypes in an executive function task in rhesus monkeys. Pharmacol. Biochem. Behav. 104, 62–68. 10.1016/j.pbb.2012.12.018 23290931 PMC3977599

[B33] Meng C. Wang D. Zhao Y. Sun J. Miao G. Chen L. (2025). Dexmedetomidine for delirium prevention in adult patients following cardiac surgery: a meta-analysis of randomized controlled trials. J. Cardiothorac. Surg. 20 (1), 110. 10.1186/s13019-025-03360-7 39891182 PMC11783797

[B34] Oto J. Yamamoto K. Koike S. Onodera M. Imanaka H. Nishimura M. (2012). Sleep quality of mechanically ventilated patients sedated with dexmedetomidine. Intensive Care Med. 38 (12), 1982–1989. 10.1007/s00134-012-2685-y 22961436

[B35] Pandharipande P. P. Pun B. T. Herr D. L. Maze M. Girard T. D. Miller R. R. (2007). Effect of sedation with dexmedetomidine vs lorazepam on acute brain dysfunction in mechanically ventilated patients: the MENDS randomized controlled trial. JAMA 298 (22), 2644–2653. 10.1001/jama.298.22.2644 18073360

[B36] Park E. A. Kim M. Y. (2019). Postoperative delirium is associated with negative outcomes and long-term mortality in elderly Koreans: a retrospective observational Study. Med. Kaunas. 55 (10), 618. 10.3390/medicina55100618 31547219 PMC6843516

[B37] Perbet S. Verdonk F. Godet T. Jabaudon M. Chartier C. Cayot S. (2018). Low doses of ketamine reduce delirium but not opiate consumption in mechanically ventilated and sedated ICU patients: a randomised double-blind control trial. Anaesth. Crit. Care Pain Med. 37 (6), 589–595. 10.1016/j.accpm.2018.09.006 30268528

[B38] Pisani M. A. Murphy T. E. Araujo K. L. Slattum P. Van Ness P. H. Inouye S. K. (2009). Benzodiazepine and opioid use and the duration of intensive care unit delirium in an older population. Crit. Care Med. 37 (1), 177–183. 10.1097/CCM.0b013e318192fcf9 19050611 PMC2700732

[B39] Reel B. Maani C. V. (2025). “Dexmedetomidine. 2023 May 1,” in StatPearls. Treasure Island (FL) (Treasure Island: StatPearls Publishing). Available online at: https://www.ncbi.nlm.nih.gov/books/NBK513303/(accessed on April 1, 2025). 30020675

[B40] Rudolph J. L. Marcantonio E. R. (2011). Review articles: postoperative delirium: acute change with long-term implications. Anesth. Analg. 112 (5), 1202–1211. 10.1213/ANE.0b013e3182147f6d 21474660 PMC3090222

[B41] Shang L. Hou M. Guo F. (2023). Postoperative application of dexmedetomidine is the optimal strategy to reduce the incidence of postoperative delirium after cardiac surgery: a network meta-analysis of randomized controlled trials. Ann. Pharmacother. 57 (3), 221–231. 10.1177/10600280221106622 35815719

[B42] Shen Q. H. Li H. F. Zhou X. Y. Yuan X. Z. (2020). Dexmedetomidine in the prevention of postoperative delirium in elderly patients following non-cardiac surgery: a systematic review and meta-analysis. Clin. Exp. Pharmacol. Physiol. 47 (8), 1333–1341. 10.1111/1440-1681.13312 32215933

[B43] Shi Z. Mei X. Li C. Chen Y. Zheng H. Wu Y. (2019). Postoperative delirium is associated with long-term decline in activities of daily living. Anesthesiology 131 (3), 492–500. 10.1097/ALN.0000000000002849 31335550 PMC6692194

[B44] Skrobik Y. Duprey M. S. Hill N. S. Devlin J. W. (2018). Low-Dose nocturnal dexmedetomidine prevents ICU delirium. A randomized, placebo-controlled trial. Am. J. Respir. Crit. Care Med. 197 (9), 1147–1156. 10.1164/rccm.201710-1995OC 29498534

[B45] Sosnowski K. Lin F. Chaboyer W. Ranse K. Heffernan A. Mitchell M. (2023). The effect of the ABCDE/ABCDEF bundle on delirium, functional outcomes, and quality of life in critically ill patients: a systematic review and meta-analysis. Int. J. Nurs. Stud. 138, 104410. 10.1016/j.ijnurstu.2022.104410 36577261

[B46] Su X. Meng Z. T. Wu X. H. Cui F. Li H. L. Wang D. X. (2016). Dexmedetomidine for prevention of delirium in elderly patients after non-cardiac surgery: a randomised, double-blind, placebo-controlled trial. Lancet 388 (10054), 1893–1902. 10.1016/S0140-6736(16)30580-3 27542303

[B47] Subramaniyan S. Terrando N. (2019). Neuroinflammation and perioperative neurocognitive disorders. Anesth. Analg. 128 (4), 781–788. 10.1213/ANE.0000000000004053 30883423 PMC6437083

[B48] Sun W. Zhao J. Li C. (2020). Dexmedetomidine provides protection against Hippocampal Neuron apoptosis and cognitive impairment in mice with Alzheimer's Disease by mediating the miR-129/YAP1/JAG1 axis. Mol. Neurobiol. 57 (12), 5044–5055. 10.1007/s12035-020-02069-z 32839917

[B49] Turan A. Duncan A. Leung S. Karimi N. Fang J. Mao G. (2020). Dexmedetomidine for reduction of atrial fibrillation and delirium after cardiac surgery (DECADE): a randomised placebo-controlled trial. Lancet 396 (10245), 177–185. 10.1016/S0140-6736(20)30631-0 32682483

[B50] Vacas S. Van de Wiele B. (2017). Designing a pain management protocol for craniotomy: a narrative review and consideration of promising practices. Surg. Neurol. Int. 8, 291. 10.4103/sni.sni_301_17 29285407 PMC5735429

[B51] van Gelder T. G. van Diem-Zaal I. J. Dijkstra-Kersten S. M. A. de Mul N. Lalmohamed A. Slooter A. J. C. (2024). The risk of delirium after sedation with propofol or midazolam in intensive care unit patients. Br. J. Clin. Pharmacol. 90 (6), 1471–1479. 10.1111/bcp.16031 38482541

[B52] Wang D. Liu Z. Zhang W. Zu G. Tao H. Bi C. (2024a). Intravenous infusion of dexmedetomidine during the surgery to prevent postoperative delirium and postoperative cognitive dysfunction undergoing non-cardiac surgery: a meta-analysis of randomized controlled trials. Eur. J. Med. Res. 29 (1), 239. 10.1186/s40001-024-01838-z 38637853 PMC11025279

[B53] Wang E. Belley-Côté E. P. Young J. He H. Saud H. D'Aragon F. (2023). Effect of perioperative benzodiazepine use on intraoperative awareness and postoperative delirium: a systematic review and meta-analysis of randomised controlled trials and observational studies. Br. J. Anaesth. 131 (2), 302–313. 10.1016/j.bja.2022.12.001 36621439

[B54] Wang D. He X. Li Z. Tao H. Bi C. (2024b). The role of dexmedetomidine administered *via* intravenous infusion as adjunctive therapy to mitigate postoperative delirium and postoperative cognitive dysfunction in elderly patients undergoing regional anesthesia: a meta-analysis of randomized controlled trials. BMC Anesthesiol. 24 (1), 73. 10.1186/s12871-024-02453-5 38395794 PMC10885557

[B55] Weerink M. A. S. Struys MMRF Hannivoort L. N. Barends C. R. M. Absalom A. R. Colin P. (2017). Clinical pharmacokinetics and pharmacodynamics of dexmedetomidine. Clin. Pharmacokinet. 56 (8), 893–913. 10.1007/s40262-017-0507-7 28105598 PMC5511603

[B56] Yoo S. H. Kang J. Kim H. J. Lee S. W. Hong M. Jung E. H. (2024). Opioid use and subsequent delirium risk in patients with advanced cancer in palliative care: a multicenter registry study. Sci. Rep. 14 (1), 6004. 10.1038/s41598-024-56675-1 38472471 PMC10933309

[B57] Zeng H. Li Z. He J. Fu W. (2019). Dexmedetomidine for the prevention of postoperative delirium in elderly patients undergoing noncardiac surgery: a meta-analysis of randomized controlled trials. PLoS One 14 (8), e0218088. 10.1371/journal.pone.0218088 31419229 PMC6697366

[B58] Zhang D. F. Su X. Meng Z. T. Li H. L. Wang D. X. Li X. Y. (2019). Impact of dexmedetomidine on long-term outcomes after noncardiac surgery in elderly: 3-year Follow-up of a randomized controlled trial. Ann. Surg. 270 (2), 356–363. 10.1097/SLA.0000000000002801 29742525

[B59] Zhuang X. Fu L. Luo L. Dong Z. Jiang Y. Zhao J. (2024). The effect of perioperative dexmedetomidine on postoperative delirium in adult patients undergoing cardiac surgery with cardiopulmonary bypass: a systematic review and meta-analysis of randomized controlled trials. BMC Anesthesiol. 24 (1), 332. 10.1186/s12871-024-02715-2 39289619 PMC11406813

